# Fiber Reshaping-Based Refractive Index Sensor Interrogated through Both Intensity and Wavelength Detection

**DOI:** 10.3390/s19112477

**Published:** 2019-05-30

**Authors:** Peng Ji, Shiru Jiang, Sang-Shin Lee

**Affiliations:** Department of Electronic Engineering, Kwangwoon University, 20 Kwangwoon-ro, Nowon-gu, Seoul 01897, Korea; prl.jipeng@gmail.com (P.J.); prl.jiangshiru@gmail.com (S.J.)

**Keywords:** refractive index sensor, single-mode fiber, ultrafast laser inscription, multimode interference, intensity variation, wavelength shift

## Abstract

A fiber reshaping-based refractive index (RI) sensor is proposed relying on both optical intensity variation and wavelength shift. The objective of this study is to completely reshape the core and to ultimately mimic a coreless fiber, thereby creating a highly efficient multimode interference (MMI) coupler. Thus, propagation modes are permitted to leak out into the cladding and eventually escape out of the fiber, depending on the surrounding environment. Two interrogation mechanisms based on both the intensity variation and wavelength shift are employed to investigate the performance of the RI sensor, with the assistance of leaky-mode and MMI theories. By monitoring the output intensity difference and the wavelength shift, the proposed RI sensor exhibits high average sensitivities of 185 dB/RIU and 3912 nm/RIU in a broad range from 1.339 to 1.443, respectively. The operating range and sensitivity can be adjusted by controlling the interaction length, which is appealing for a wide range of applications in industry and bioscience research.

## 1. Introduction

Refractive index (RI) sensors have been the subject of a significant number of investigations with the intention of measuring the RI and concentration of liquids or gases for diverse applications in industry and bioscience research. Among these, fiber-optic approaches have long been actively pursued because of their unparalleled merits, which include compact size, light weight, low cost, high sensitivity, rapid response time, electromagnetic interference immunity, durability to harsh environment, and chemical neutrality [1,2]. Thus far, a substantial number of fiber-optic designs for RI sensing have been presented. These include tapered multimode fiber (MMF) tips [3,4]; bent fibers incorporating a plastic MMF with a large numerical aperture (NA) [5], standard single-mode fibers (SMFs) with a reduced cladding diameter [6], and dual-channel SMF bending [7]; fiber Bragg gratings (FBGs), including long-period gratings, surface FBGs, macro-bent FBGs, and tilted moiré FBGs [8,9,10,11]; side-polished or D-shaped fibers [12,13,14,15]; waist-deformed fiber tapers fabricated by heat or chemical tapering [16,17,18]; heterostructures formed by the splicing of hetero-core fibers, including MMF-SMF-MMF [19], SMF-MMF-SMF [20], SMF-tapered claddingless fiber-SMF [21], SMF-hole-assisted dual-core fiber-SMF [22], and cascaded single-mode-no-core-hollow-core-no-core-single-mode structures [23].

In particular, for the heterostructure-based designs, in order to facilitate the interaction between the core waveguide and surrounding medium, the cladding of the sensor portion may be fully or partly removed through additional processes, such as chemical etching [24] or femtosecond laser ablation [25]. To avoid the splicing process, an approach of reducing the average RI of the fiber core was recently reported by introducing a line of negative RI modification, which partly overlaps with the fiber core over an interaction length of mm scale. Considering there are a multitude of similar interference dips for each transmission spectrum, it might be notably demanding to directly track individual dips. To surmount this issue, additional data processing (e.g., a discrete Fourier transform) should inevitably be required [26]. Moreover, surface plasmon resonance has been used to further improve resolution and sensitivity by coating thin metal layers on the surface of the sensor [27,28,29]. Alternatively, various types of microstructure have also been developed, relying on femtosecond laser-induced microfluidic structures [30,31], Mach-Zehnder interferometers [32,33], and Fabry-Perot interferometers [34,35]. In general, sensing mechanisms, resorting to either optical intensity variation or wavelength shift, may underpin the operation of the RI sensor. The performance, in terms of sensing range and average sensitivity, is shown for the previously reported RI sensors described above in Appendix A.

Ultrafast laser inscription (ULI) has been well established as one of the most efficient micromachining techniques, providing a powerful and flexible tool for the fabrication of three-dimensional photonic devices in dielectric materials [36]. Focused ultrashort laser pulses are known to provide the ability to locally modify the RI of the material through nonlinear energy transfer processes [37,38]. The pulse-induced permanent change in RI can either be positive or negative depending on the increase or decrease in RI of the interacting medium [38,39,40,41]. Positive index modification, which can be relatively easily realized with a low irradiation power, is common for the creation of optical waveguides in amorphous materials, e.g., in most glasses [42,43,44]. The fabrication utilizing a negative regime is mainly used for a depressed cladding structure. ULI-fabricated depressed structures with customized cross-sections have been reported in glass and crystalline materials, such as Tm^3+^:ZBLAN glass [45], Ho^3+^:ZBLAN glass [46], phosphate glass [47], Cr:ZnSe [48], Cr:ZnS [49], Tm^3+^:YAG [39], Cr^4+^:YAG [50], Nd^3+^:YAG [51], LiNbO_3_ [52,53], and sapphire [54,55]. For the optical waveguide, its dimensions, number of propagation modes, amount of RI change, and NA can be altered by tailoring the interacting materials and exposure conditions. Hence, ULI can be regarded as a prime potential technique to modify the propagation and interaction of the modes in an optical fiber in a convenient manner.

In this study, a fiber reshaping-based RI sensor featuring a high sensitivity is proposed and designed, allowing for both direct detection of intensity variation and wavelength shift simultaneously. The proposed sensor simply engages a section of completely reshaped SMF core, which might be implemented via the ULI-induced RI modification. Optical confinement associated with the core is destroyed to excite higher-order cladding modes, which are vulnerable to external perturbations. Leaky-mode and multimode interference (MMI) theories are primarily utilized to investigate the transmission characteristics of the sensor. The impact of the interaction length on both the intensity- and wavelength-based interrogation has been discussed. To verify the feasibility and flexibility of the RI sensor, several SMFs with different core diameters and NAs are considered, with the help of a simulation tool, RSoft BeamPROP (Synopsys).

## 2. Configuration of the Proposed Fiber-optic RI Sensor

**Figure 1:**
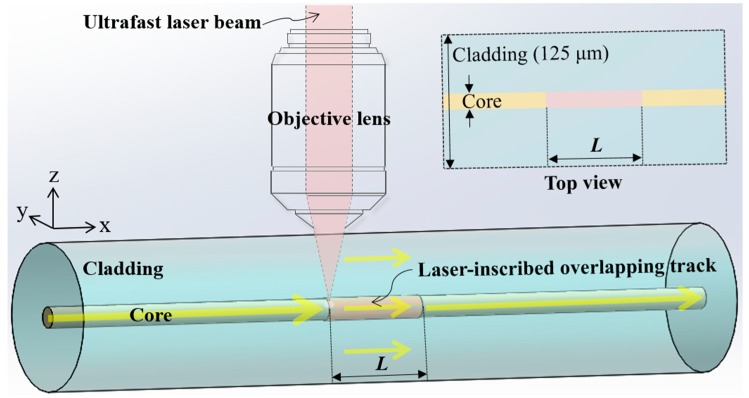
Schematic of the proposed fiber-optic RI sensor, with a section of the SMF core completely reshaped. Inset (top-right corner): top view observed in the *x*-*y* plane of the core including the reshaped section of length *L* in pink.

The aim of this work is to completely reshape a section of the SMF core and ultimately to mimic a conventional SMF-MMF-SMF architecture, thus substantially reinforcing the optical interaction between the fiber and the surrounding medium. As illustrated in Figure 1, the proposed sensor consists of only a section of core (pink), reshaped by utilizing the ULI-induced RI modification pertaining to the negative regime. Admitting that the current work presented in this paper is preferentially focused on the theoretical design of a RI sensor, its fabrication might be realized by translating the fiber under the focused femtosecond laser beam exhibiting a visible central wavelength, as in the case of ref. 26 in which the central wavelength, pulse energy, repetition rate, and translating speed are 520 nm, 3 μJ, 200 kHz, and 0.2 mm/s, respectively. The corresponding exposure time is 75 s for a RI senor with an interaction length of 15 mm. The pulsed laser beam could be delivered through a chain of turning mirrors, then focused on the fiber via a microscope objective, as shown in our previous report [56]. The induced overlapping track is designed to be located at the center of the fiber and to be concentric with the core, exhibiting the same dimensions as those of the core. The reshaped segment serves as a sensing region with an interaction length *L*, which is basically equal to the re-imaging distance pertaining to a highly efficient MMI coupler [19,24].

The sensor is implemented in the form of an uninterrupted transmission line, capitalizing on a single SMF with a standard cladding diameter of 125 μm. The fiber is jacketless with its coating stripped off, allowing the cladding to be in direct contact with the surrounding medium whose RI is to be measured. Under this condition, the cladding in the sensing region is designed to guide light and function as a quasi-core, while the outer environment with an unknown RI acts as a quasi-cladding.

The reshaping plays the role of substantially suppressing the RI contrast between the core and cladding to form a “coreless” fiber, whereby the light (indicated by yellow arrows) traveling in the core is mostly supposed to leak into the cladding. A portion of the cladding modes will further leak outside the fiber, and the power of the transmitted light accordingly changes depending on the RI of the medium surrounding the fiber, in accordance with the self-imaging phenomenon [57]. One intriguing feature of the proposed sensor is that the transmission peak wavelength shift induced by the MMI can be used to monitor the RI of the surrounding medium as well, enabling more flexible measurements. As the sensor requires no bending, splicing, or polishing of the fiber, the proposed design is anticipated to offer an additional benefit in terms of robust mechanical strength, which is beneficial for the cleaning process. Consequently, the sensor is deemed to be reusable through a simple cleaning step without destroying the sensor. The design procedure for the proposed RI sensor and its expected performance under different structural configuration are discussed in Appendix B.

## 3. Mechanism for RI Sensing

As aforementioned, when light is launched into the SMF possessing a fully reshaped core, higher-order cladding modes will be excited. Therefore, the propagation modes will leak into the cladding, and interference between these cladding modes will occur while part of the light escapes out of the fiber depending on the external perturbations. After going through the well-developed “coreless” region, some of the propagation modes will recouple back to the SMF core.

For the optical intensity-based interrogation, RI sensing is realized in accordance with the leaky-mode theory that involves evanescent wave interaction between the transmission light wave and surrounding environment [19]. An increase in the RI for a given surrounding medium leads to variations in the critical angle. As per the well-known Snell’s law, the critical angle, given by $\sin^{-1}\left(n_{sur}/n_{clad}\right)$, is proportional to the surrounding RI (*n_sur_*) in the sensing area, where *n_clad_* is the RI of the SMF cladding. When total internal reflection condition is no longer satisfied, the light wave reflected at the outermost cladding interface may succeed in escaping the fiber, indicating a *n_sur_*-dependent mode leakage into the surrounding medium.

When it comes to the wavelength-shift-based interrogation, the underlying principle of the proposed design is based on MMI theory. Owing to the interference between the multiple higher-order modes, the transmission spectra of the proposed sensor could include several interference peaks at different wavelength ranges. Considering the circular symmetry of the fundamental mode of the SMF, the input light is assumed to have a field distribution of *E*(*λ*, 0), and only the eigenmodes LP_0m_ will be excited. Defining the field profile of LP_0m_ as *F_m_*(*λ*) and neglecting the small amount of radiation, the field along the sensor at the propagation distance *x* is calculated by using the expression [58]:

(1) $$E\left(\lambda ,\;x\right)=\overset{M}{\underset{m=1}{\sum }}c_{m}F_{m}\left(\lambda \right)\exp\left(i\beta_{m}x\right).$$

The number of excited LP_0m_ modes in the “coreless” region is:

(2) $$M=\frac{2r}{\lambda }\sqrt{n_{clad}^{2}-n_{sur}^{2}}\;,$$

while the transmission intensity is obtained by using the overlap integral method [58]:

(3) $$P\left(x\right)=10{log}_{10}\left\{{\left[\overset{M}{\underset{m=1}{\sum }}c_{m}^{2}exp\left(i\beta_{m}x\right)\right]}^{2}\right\}.$$

Here, *c_m_* and *β_m_* are the excitation coefficient and propagation constant of each eigenmode, respectively; *λ* is the wavelength in free space, and *r* is the radius of the SMF cladding. If the accumulated phase difference induced by *β_m_* between two eigenmodes is an integer multiple of 2π, their interference will play a dominant role in giving rise to the MMI pattern. The peak wavelength of the transmission spectrum is approximately expressed as:

(4) $$\lambda_{p}=\frac{16Kn_{eff}r^{2}}{\left(m-n\right)\left[2\left(m+n\right)-1\right]L},$$

where *n_eff_* is the effective RI of the excited cladding mode and determined by the difference between *n_sur_* and *n_clad_* [7,24,25]; *m* and *n* are the mode orders, while *K* is an integer [21]. It is known that the difference between *n_sur_* and *n_clad_* decreases as *n_sur_* increases when *n_sur_* < *n_clad_*, resulting in a larger Goos-Hänchen shift; thus, *n_eff_* increases under the same condition [21]. Consequently, red-shifting of the peak wavelength of the transmission spectrum occurs when the surrounding RI increases. In addition, the interaction length affects the interference between the eigenmodes, exhibiting red-shifts in the peak wavelength for shorter-length sensors.

## 4. Characteristics of the Proposed RI Sensor

**Figure 2:**
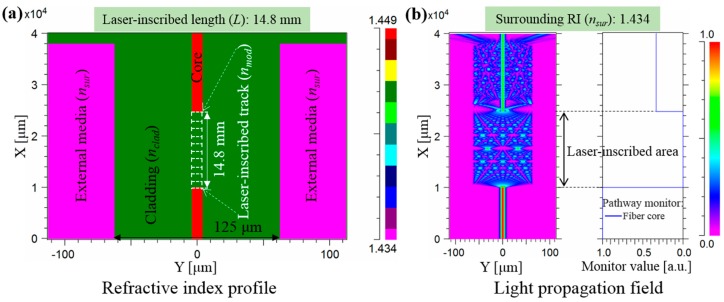
(**a**) Architecture and the corresponding index profile of the proposed RI sensor. (**b**) Light propagation behavior for the reshaped SMF for a surrounding medium of *n_sur_* = 1.434.

For the numerical calculation, a commercially available beam propagation method tool (RSoft BeamPROP) was adopted to model and assess the light propagation behavior. Figure 2a shows the structure of the proposed RI sensor and its index profile. The measurement setup which can be engaged for practical testing is depicted in Appendix C. A standard step-index SMF is considered for the embodiment of the sensor, for which the core and cladding are 8.2 μm and 125 μm in diameter, respectively, and the corresponding RIs are *n_core_* = 1.449 and *n_clad_* = 1.444 at the wavelength of 1550 nm. The laser-induced track fully overlaps the core, playing the role of optically reshaping the core to form a “coreless fiber”. The length of the reshaped core that serves as the sensing region is *L* = 14.8 mm, which is approximately equal to the re-imaging distance. Based on the previous femtosecond laser-writing tests [26,44], it is presumed that the RI of the modified core is uniformly the same as that of the cladding (*n_mod_* = *n_clad_*), which corresponds to a negative RI change of −0.005. As indicated in Figure 2b, the fundamental fiber mode at the 1550 nm wavelength is launched into the SMF along the *x*-direction, and the pathway monitor is placed along the propagation direction. When light from the lead-in SMF arrives at the “coreless” territory, the light largely leaks out into the cladding in the presence of the fully reshaped core. For a given surrounding medium of *n_sur_* = 1.434, approximately 33.3% of the incident power recouples back to the SMF core and then remains constant after the sensing region, while the remaining light propagates as cladding modes and leaks out the fiber at the boundary between the surrounding media and lead-out SMF. As anticipated, the surrounding RI can therefore be determined by observing the output power from the fiber.

**Figure 3:**
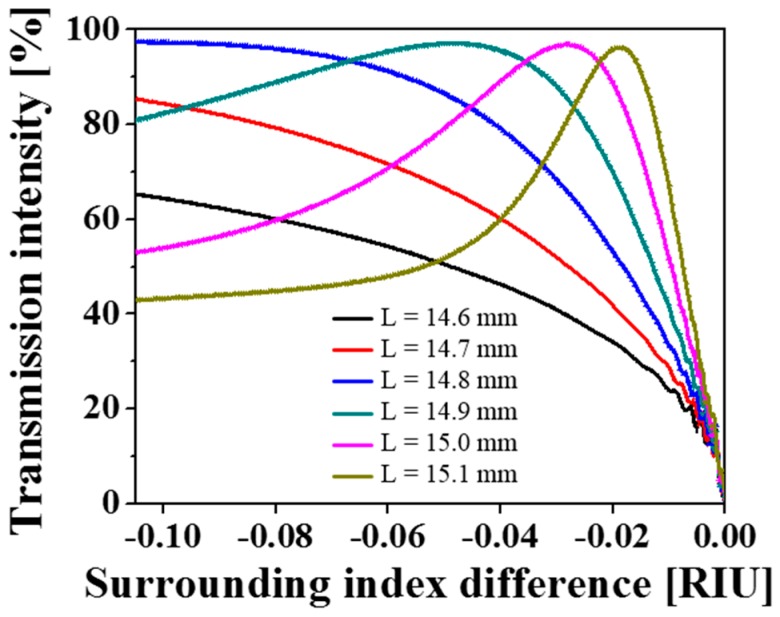
Calculated transmission curves with respect to the surrounding RI for different interaction lengths.

For the optical intensity-based interrogation, the calculated transmission for different interaction lengths is plotted in Figure 3. The relative change in the surrounding RI, defined as $\Delta n_{sur}=n_{sur}-n_{clad}$, is particularly used for the following demonstration. The transmission responses are obtained by launching the fundamental mode at a wavelength of 1550 nm as the input field. Δ*n_sur_* ranges from −0.105 to 0 with a resolution of 0.001, as the RI of liquids is typically no less than 1.340. The optical intensity varies remarkably, hinging on the surrounding RI. In the cases of *L* = 14.6 mm, 14.7 mm, and 14.8 mm, the transmission intensity increases monotonously, as the surrounding index decreases from Δ*n_sur_* = 0 to −0.105. However, for *L* > 14.8 mm, the non-monotonic peak intensities turn out to be caused by the MMI, which is governed by the propagation constant and the phase difference between each mode. Furthermore, the transmission intensity drops sharply to near zero owing to the mode leakage when Δ*n_sur_* approaches zero. The slope of the transfer curves is more pronounced for sensors with longer interaction lengths, demonstrating higher sensitivity, while its monotonic range of measurable indices is narrower.

Here, the sensitivity alludes to the change in transmission intensity for a unit change in RI of the surrounding medium (RIU: RI unit). The average sensitivities corresponding to different sensing ranges of the sensor, as determined via intensity-based interrogation, are tabulated in Table 1. The dynamic range and sensitivity can be readily tailored for a specific application by varying the interaction length.

**Figure 4:**
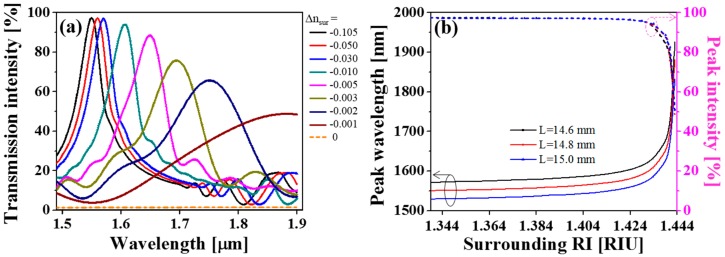
(**a**) Transmission spectra for the sensor under different surrounding RIs for the case of *L* = 14.8 mm. (**b**) Peak wavelength and peak intensity of the transmission spectra as a function of the surrounding RI for the sensors with *L* = 14.6 mm, 14.8 mm, and 15.0 mm.

Meanwhile, with the intention of providing insight into the influence of the MMI, the transmission spectra were examined for incident wavelengths scanning from 1.5 μm to 1.9 μm for *L* = 14.8 mm. As depicted in Figure 4a, the transmission spectra were normalized with respect to the input light power. The transmission spectra at different surrounding RIs reveal that the interference peak shifts noticeably and monotonically toward longer wavelengths (red-shifts) as $\left|\Delta n_{sur}\right|$ decreases, especially for $\left|\Delta n_{sur}\right|<0.030$, which is consistent with the prediction [21]. In this regime, high sensitivity and high resolution can be achieved concurrently. For instance, the wavelength shifts are 10.5 nm and 1.5 nm for the cases of Δ*n_sur_* = −0.00100 ± 10^−5^ and −0.00300 ± 10^−5^, respectively. The peak intensity reaches an almost steady output when Δ*n_sur_* < −0.030, providing a slow variation in transmission intensity as the light guided by the fiber rarely radiates. In contrast, the peak intensity is observed to decline dramatically when Δ*n_sur_* approaches zero, by virtue of the enhanced evanescent field and resulting mode leakage. For the case of Δ*n_sur_* = 0, a negligible transmission intensity can be recoupled back to the core of the SMF, and no interference peak is obtained. This might be attributed to the leakage of all the forward-propagating modes into the external surroundings. On top of the reduced optical intensity, the bandwidth of the transmission spectrum broadens with increasing surrounding RI to deteriorate the Q-factor. This is caused by the reduced number of excited modes according to Equation (2), resulting in weakened MMI resonance in the “coreless” fiber section [24,59].

Moreover, as plotted in Figure 4b, the peak wavelength red-shifts with shorter interaction lengths, as predicted [21], entailing slight changes in the sensitivity. The sensitivity refers to the change in the wavelength shift for a unit change in RI of the surrounding medium. The average sensitivities and operating ranges of the sensor, which is interrogated through the wavelength shift, are given in Table 2. The achieved sensitivities in average are much higher than the conventional heterostructure-based designs [19,21,22,23,24,25,26].

**Figure 5:**
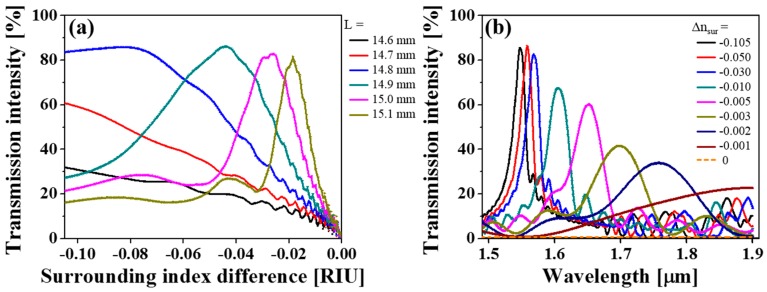
(**a**) Calculated transmission curves for the proposed sensor as a function of the surrounding index difference at *λ* = 1550 nm under different interaction lengths and (**b**) its spectral responses based on an SMF (980-HP) with *L* = 14.8 mm.

**Figure 6:**
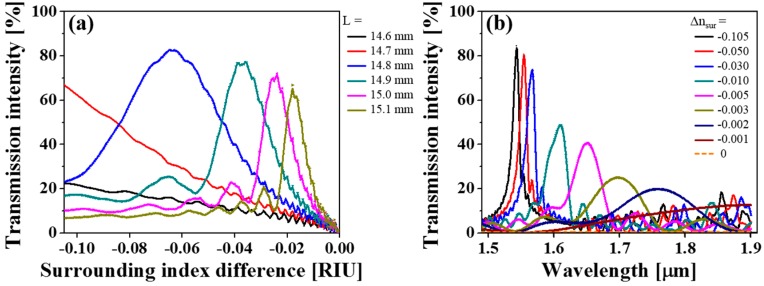
(**a**) Calculated transmission curves for the proposed sensor as a function of the surrounding index difference at *λ* = 1550 nm under different interaction lengths and (**b**) its spectral responses based on an SMF (UHNA1) with *L* = 14.8 mm.

In a bid to confirm the feasibility and flexibility of the proposed mechanism for RI sensing, a set of SMFs with different core diameters and NAs were explored, in the aspects of both intensity- and wavelength-based interrogations. Figure 5 and Figure 6 plot the calculated transmission curves at the wavelength of 1550 nm and the spectral responses for the proposed sensor, which taps into an SMF of 980-HP (3.6-μm core diameter, NA = 0.20) and UNHA1 (2.5-μm core diameter, NA = 0.28) [60], respectively. The results are consistent with the calculations based on the standard step-index SMF.

For the intensity-based interrogations, the peak transmission declines and its monotonic range of measurable indices becomes narrow, yielding quasi-monotonic ranges of Δ*n_sur_* > −0.083 and −0.063 for the SMFs 980-HP and UNHA1, respectively, when *L* = 14.8 mm. This may be interpreted as a result of poor light coupling between the heterostructures, and consequently a smaller signal-to-noise ratio, resulting from the smaller core diameter. For the wavelength-based interrogation, however, the transmission spectra and corresponding peak wavelength remain almost consistent with the previous results, despite the reduced optical intensities. Furthermore, it seems that the sensitivity has been a little alleviated; average sensitivities in the wavelength shift of 3356 nm/RIU and 3413 nm/RIU over the range from Δ*n_sur_* = −0.105 to −0.001 are demonstrated. Therefore, it can be concretely corroborated that the proposed RI sensing can be realized, either by monitoring the optical intensity variation or wavelength shift for different SMFs, allowing for flexible design and measurement.

## 5. Conclusions

In summary, a highly sensitive fiber-optic RI sensor, exploiting an SMF with an optically reshaped core, has been proposed and designed. RI detection can be realized by either monitoring the output intensity alteration or the peak wavelength shift. Leaky-mode and MMI theories are primarily involved in investigating the transmission behavior of the sensor. The impact of the interaction length on the sensor performance was analyzed particularly, corroborating an excellent flexibility in tuning the operating range and sensitivity. For a standard step-index fiber with a completely reshaped core of 14.8 mm in length, ultrahigh average sensitivities of 185 dB/RIU and 3912 nm/RIU were obtained in the range of 1.339−1.443 for the intensity- and wavelength-based interrogations, respectively, which are much higher compared to the conventional heterostructure-based designs. The calculations also indicate that the proposed sensing scheme can be applied to different types of SMFs, regardless of their core diameter and NA. The proposed RI sensor has potential for use in a wide range of possible applications for measuring the RI and concentration of liquids or gases in industry and bioscience.

Our future work will focus on the correction and improvement of the negative modification in RI. Currently, the experimental demonstration is still under investigation due to the fact the entirely negative modification of RI in fusion silica-based SMFs is difficult to achieve via our own ultrafast laser writing system [35,43]. Special SMFs made of crystal materials, as well as visible light-based femtosecond laser pulses [26], are under consideration to implement ULI-based fiber reshaping. In addition, LiNbO_3_-based ULI for the negative modification in RI will be an interesting and practical topic.

## Figures and Tables

**Table 1:** Sensing performance interrogated through intensity detection at *λ* = 1550 nm.

Sensing Range	Average Sensitivity (dB/RIU)		
	*L* = 14.6 mm	*L* = 14.8 mm	*L* = 15.0 mm
0.001 (Δ*n_sur_* = −0.001 to 0)	6949	5363	6433
0.005 (Δ*n_sur_* = −0.005 to 0)	2261	2646	2780
0.010 (Δ*n_sur_* = −0.010 to 0)	1327	1476	1669
0.050 (Δ*n_sur_* = −0.050 to 0)	330	378	Non-monotonic
0.105 (Δ*n_sur_* = −0.105 to 0)	168	185	Non-monotonic

**Table 2:** Sensing performance for the interrogation through wavelength detection.

Sensing Range	Average Sensitivity (nm/RIU)		
	*L* = 14.6 mm	*L* = 14.8 mm	*L* = 15.0 mm
0.001 (Δ*n_sur_* = −0.002 to −0.001)	145,000	131,000	110,000
0.005 (Δ*n_sur_* = −0.006 to −0.001)	53,800	49,400	43,800
0.010 (Δ*n_sur_* = −0.011 to −0.001)	30,200	28,000	25,300
0.050 (Δ*n_sur_* = −0.051 to −0.001)	6900	6440	5860
0.104 (Δ*n_sur_* = −0.105 to −0.001)	3423	3912	2913

**Figure A1:**
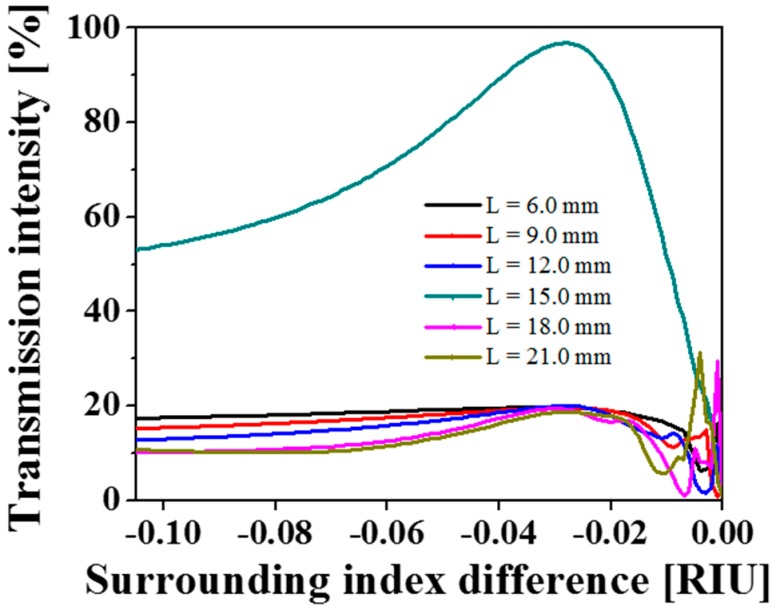
Transmission curves with respect to the surrounding RI for different interaction lengths. [Note that the results are all calculated for the case of a fully modified fiber core with Δ*n_mod_* = −0.005 at *λ* = 1550 nm.]

**Figure A2:**
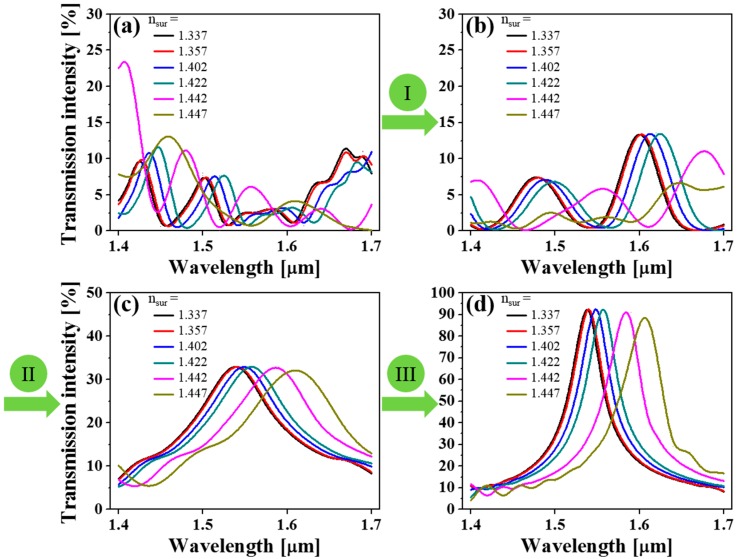
Transmission spectra for an MMI-based RI sensor under four different structural configurations. (**a**) The design incorporating a partly modified fiber core in width and *L* << *L*’. (**b**) A fully modified fiber core for *L* << *L*’. (**c**) A fully modified fiber core under a tenuous modification intensity and *L* << *L*’. (**d**) The proposed case pertaining to a fully modified fiber core under a tenuous modification intensity and *L* ≈ *L*’. Evolution from I to III leads to a complete modification of the fiber core, reducing the modification intensity from −0.013 to −0.005, and increasing the interaction length up to the re-imaging distance (*L* ≈ *L*’).

**Figure A3:**
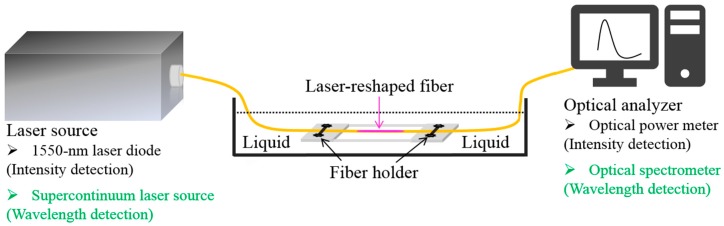
Configuration of the measurement setup which could be used for practical testing of the proposed RI sensor.

## Appendix A

For the previously reported RI sensors, the performance, including the sensing range and average sensitivity, is presented in Table A1 below.

**Table A1:** Performance comparison for the previously reported RI sensing.

Sensing Mechanism	Sensing Range	Δ*n*	Average Sensitivity	Reference
Intensity interrogation	1.360–1.459	0.099	~ 101 ^1^ dB/RIU	[3]
Intensity interrogation	1.3314–1.4156	0.0842	< 28 ^1^ dB/RIU	[4]
Intensity interrogation	1.357–1.376	0.019	< 25 ^1^ dB/RIU	[5]
Intensity interrogation	1.4586–1.5396	0.0810	~ 170 ^1^ dB/RIU	[6]
Wavelength shift	1.3403–1.3726	0.0323	245 nm/RIU	[7]
Wavelength shift	N/A	0.012	2.3 nm/RIU	[8]
Wavelength shift	1.400–1.450	0.050	< 16 nm/RIU	[9]
Wavelength shift	1.3330–1.3785	0.0455	165.9 nm/RIU	[10]
Wavelength shift	1.390–1.445	0.055	545.9 nm/RIU	[11]
Intensity interrogation	1.332–1.471	0.139	< 25 ^1^ dB/RIU	[13]
Intensity interrogation	1.33–1.44	0.11	864 %/RIU	[14]
Wavelength shift	1.333–1.398	0.065	4122 nm/RIU	[15]
Wavelength shift	1.410–1.442	0.032	~ 3188 ^1^ nm/RIU	[16]
Wavelength shift	1.3574–1.3686	0.0112	68.5 nm/RIU	[17]
Wavelength shift	1.333–1.413	0.080	1.5 nm/RIU	[18]
Intensity interrogation	1.412–1.456	0.044	< 318 ^1^ dB/RIU	[19]
Wavelength shift	1.333–1.399	0.066	163.8 nm/RIU	[21]
Wavelength shift	1.335–1.395	0.060	353.9 nm/RIU	[22]
Intensity interrogation	1.334–1.384	0.050	367.9 dB/RIU	[23]
Wavelength shift	1.342–1.437	0.095	~ 537 ^1^ nm/RIU	[24]
Wavelength shift	1.3373–1.4345	0.0972	610 nm/RIU	[25]
Wavelength shift	1.3333–1.4513	0.118	~ 1270 ^1^ nm/RIU	[26]
Wavelength shift	1.346–1.388	0.042	2323.4 nm/RIU	[27]
Wavelength shift	1.27–1.45	0.18	5653.6 nm/RIU	[28]
Wavelength shift	1.3000–1.3350	0.0350	~ 12,570 ^1^ nm/RIU	[29]
Wavelength shift	1.408–1.426	0.018	~ 9.9 nm/RIU	[30]
Intensity interrogation	1.333–1.473	0.140	18 dB/RIU	[31]
Wavelength shift	1.3330–1.33801	0.00501	12,162.0 nm/RIU	[32]
Wavelength shift	1.315–1.442	0.127	~ 945 ^1^ nm/RIU	[33]
Wavelength shift	1.333–1.390	0.057	1147.5 nm/RIU	[34]
Intensity interrogation	1.333–1.435	0.102	~ 392 ^1^ dB/RIU	[35]

^1^ The data were not directly provided in the corresponding reports but derived by the authors of the present paper.

## Appendix B

The proposed RI sensor has been elaborately designed to improve the performance as follows. For the intensity-based interrogation, light beam spreads and converges while propagating in the multimode region in accordance with the well-known self-imaging [57]. The optical intensity at the self-imaging point is varying remarkably hinging on the propagation constant of each mode, which is determined by the RI of the fiber surrounding medium. As shown in Figure A1, the maximum transmission efficiency (96.8%) is achieved for *L* = 15.0 mm, corresponding to the re-imaging distance (*L*’), indicating that the overall optical losses induced by the propagation in the “coreless” fiber section is negligible, and high intensity contrast can be realized simultaneously. Therefore, the MMI-based RI sensors are basically designed in a way that its interaction length is nearly equal to the re-imaging distance, which is defined as the propagation distance leading to a maximum coupling efficiency.

In practice however, the ultrafast laser-induced index modification, as well as the effective width and length of the created waveguide may be practically different with the theoretical design. Figure A2 shows the transmission spectra and corresponding evolutions of an MMI-based RI sensor for the cases of (a) a design incorporating a partly modified fiber core in width and *L* << *L*’, (b) a fully modified fiber core for *L* << *L*’, (c) a fully modified fiber core under a tenuous modification intensity and *L* << *L*’, and (d) the proposed scheme pertaining to a fully modified fiber core under a tenuous modification intensity and *L* ≈ *L*’.

For the wavelength shift-based interrogation, the interference caused by a group of lower-order modes (sidelobes) are substantially suppressed from Figure A2a–c, where dominant interference modes are suppressed by drawing upon a uniform RI profile and tenuous RI modification in the “coreless” fiber region. In addition, as displayed in Figure A2d, the efficiency for each transmission spectrum is significantly boosted, with the sidelobes further suppressed by increasing the interaction length to the re-imaging distance, leading to a negligible optical propagation loss of the RI sensor.

## Appendix C

Figure A3 describes the configuration of a measurement setup that could be used for practical testing of the proposed RI sensor. For the intensity-based interrogation, a highly efficient 1550-nm laser diode (e.g., 3CN00410DY, Alcatel Optronics) may be used as a light source, and the transmission intensity could be detected with an optical power meter (e.g., S122C; Thorlabs, Dachau, Germany). In the case of wavelength shift-based case, a scheme consisting of a supercontinuum laser source (e.g., SuperK Compact, NKT Photonics, Birkerød, Denmark) and a broadband optical spectrometer (e.g., NIRQuest512-2.5, Ocean Optics, Dunedin, FL, USA) may be employed for the purpose of characterizing the transmission spectrum under different surrounding RIs. In view of the relatively high output power of the supercontinuum laser and the affordable optical loss induced by ULI, a distinct transmission spectrum will be obtained. It is expected that the repeatability of the measurement from different sensors is keenly related to the accuracy of the fabrication system and measurement setup [4]. The accuracy for RI sensing is keenly related to the accuracy and resolution of the measurement setup [4,34]. In addition, measurement accuracy might be further improved by increasing the number of repeated measurement times [23].

Noting part of the cladding modes is designed to leak out of the fiber, the real-time response of the transmitted light depending on the RI of the surrounding environment could be monitored. The response time of the proposed RI sensor crucially hinges on that of the testing system, which is typically 200 ms for intensity-based detection with a digital optical power meter (1830-C, Newport, Irvine, CA, USA), and 1–200 ms for wavelength-based detection with a modular spectrometer (NIRQuest512-2.5, Ocean Optics, Dunedin, FL, USA).
